# Indigenous Microbiota to Leverage Traditional Dry Sausage Production

**DOI:** 10.1155/2021/6696856

**Published:** 2021-01-30

**Authors:** Noelia Zulema Palavecino Prpich, Germán Edgardo Camprubí, María Elisa Cayré, Marcela Paola Castro

**Affiliations:** ^1^Laboratorio de Microbiología de Alimentos, Universidad Nacional del Chaco Austral (UNCAus), Comandante Fernández 755, Presidencia Roque Sáenz Peña, 3700 Chaco, Argentina; ^2^Consejo Nacional de Investigaciones Científicas y Técnicas (CONICET), C1425FQB Buenos Aires, Argentina; ^3^Facultad de Ingeniería, Universidad Nacional del Nordeste (UNNE), Las Heras 727, Resistencia, 3500 Chaco, Argentina

## Abstract

The main issue addressed in this review is the need for innovation in the artisanal production of dry fermented sausages—leveraging rather than discarding tradition, together with some practical strategies available to achieve it. Throughout the text, emphasis is placed on the autochthonous microbiota responsible for the identity and unique sensory characteristics of these products. The available strategies to introduce innovation in this manufacturing process rely on metabolic flexibility of microbial strains. In this sense, this review evaluates the application of several tools aimed at improving the quality and safety of artisanal dry fermented sausages focusing on the microbial community role. The most studied alternatives to enhance dry sausage production comprise the use of autochthonous starter cultures—including functional and/or probiotic strains, the production of bacteriocins, and the generation of bioactive peptides, which have been thoroughly covered herein. The purpose of this work is to review recent research about novel different strategies available for food technologists to improve safety and quality in the manufacture of dry fermented sausages. Additional support strategies—quality product registers and innovation through tradition—have been suggested as complementary actions towards a successful introduction of indigenous microbial communities into traditional dry sausage production.

## 1. Introduction

Fermented meat products have been consumed for centuries throughout the world and constitute one of the most important types of food [1]. Their tradition has originated in the Mediterranean countries during Roman times [2], and their production was later extended to Germany, Hungary, and other countries, including the United States, Argentina, and Australia [3, 4]. There is a wide variety of dry fermented products on the global market as a consequence of variations in the raw materials, formulations, and manufacturing processes, which come from the habits and customs of the different countries and regions. They are closely connected to the culture, heritage, and local identity of a given population, having a strong symbolic value. Regardless of their origin, fermented sausages can be defined as meat products that comprise a stuffed mixture of pork and/or beef, fat, salt, and nitrate and/or nitrite, including eventually sugar and different spices. Albeit less frequently, formulations can include poultry, lamb, goat, horse, camel, ostrich, and game meats [5–9].

In Europe, fermented sausages can be divided into Northern and Mediterranean types [10]. Northern products are generally semidry sausages with pH below 5, while Mediterranean products are dry sausages with long ripening and drying processes,and pH values of 5.3–6.2 [4]. On the other hand, traditional sausages found in the American market are semidry sausages, fermented rapidly at relatively high temperatures, with a short drying period leading to pH values below 5 and very different organoleptic characteristics [4]. Latin American countries also produce fermented sausages; their manufacture has a long history in Argentina, Brazil, and Uruguay, mainly due to Spanish and Italian traditions, as well as to the quality and availability of beef meat. Asian and African countries also provide different types of fermented sausages, with ingredients and procedures that differ substantially with those described above [11].

The stability of fermented meat products is mainly determined by acidification—brought about by lactic acid bacteria—and water activity (*a*_w_) reduction in the course of curing and drying. In addition, biochemical and physicochemical changes occur as a result of the interactions among microorganisms, meat, fat, and processing technology; the combined effect of these factors is what produces the wide range of available fermented sausages. The development of the characteristic flavor, aroma, and texture relies on biochemical and physicochemical reactions in which several types of bacteria, yeast, and fungi species interact within the meat matrix and its surface. Lactic acid bacteria (LAB) and coagulase-negative cocci (CNC)—including both micrococci and coagulase-negative staphylococci (CNS)—are the most predominant groups in meat spontaneous fermentation [12]. Besides, filamentous fungi and yeasts exert a protective-like effect due to the formation of a superficial film preventing from excessive dehydration and oxidation of the lipid fraction due to oxygen and light [13].

Small-scale facilities persist on traditional production methods where spontaneous fermentation is the leading process. When fermentation depends on the in-house flora, microorganisms come from the meat itself and the surrounding environment supplying particular—yet heterogeneous—characteristics to the product. Industrial development has led to the use of commercial starter cultures to standardize and control sausage manufacturing; however, these cultures are not always able to reproduce especial flavors and features of artisanal dry sausages. A half-way between standardization and traditional methods could be the introduction of starter cultures especially designed using autochthonous strains isolated from the facilities [14].

Artisanal and traditional products have grown in popularity, with a return to food consumption with local identity [15]. Traditional products became more attractive largely because consumers considered them more “natural.” That perception gives the food an identity that, in turn, engenders a certain familiarity [16]. In this sense, traditional fermented dry sausages are closely connected to the culture, heritage, and local identity of a given population. They also have a strong symbolic value and contribute to the sustainability and development of rural areas [17]. Thus, this trend could be used as a development strategy for regional economies.

Within the current global economic framework, the need to add value to food products—while preserving authenticity and traditional features—is a must. Regarding dry fermented sausages, it involves not only those attributes related to their general appearance and taste but also the ones pertaining to nutritional aspects. Nowadays, health-related issues are the cornerstone of public concern about food. Consequently, salt and fat reductions have also been reviewed in this study—outside the microbial scope—in order to attend this need. The extraction of certain meat proteins by salt, leading to the emulsification of fat globules, depends on the bacterial acidification process that causes protein denaturation; consequently, variations in salt or fat concentrations would concomitantly affect microbial interactions within the meat matrix.

In addition, several technological innovations are being introduced into what are considered traditional meats. These innovations are meant to reduce production time, energy, waste, and costs as well as to meet standards of production and safety [15]. Raising the standards of local products entails quality improvement. It is noteworthy that quality is a complex concept not only based on sensory properties but also on less tangible factors, such as “traditional” and “natural” characteristics. Innovations based on the reemergence of tradition require quality guarantees and careful labelling and contribute thereby to meet legal requirements, to indicate differentiation, and to orientate and reassure consumers.

## 2. Indigenous Microbiota and Starter Cultures

In traditional sausages, the fermentation is known to rely on natural contamination by environmental microbiota occurring during slaughtering and manufacturing, being the specific composition of the “house microbiota” responsible for the distinctive qualities of artisanal products from small-scale facilities [18, 19]. Although this autochthonous microbiota plays a major role on flavor, texture, quality, and safety of the final product, the high variability in bacterial amount and species may induce quality problems due to lack of normalization and/or homogenization.

Consequently, the introduction of starter cultures in the manufacture of fermented meat products turns into a necessary implementation in order to guarantee food safety and to standardize the final product attributes [20]. Commercial starter cultures do not offer much flexibility for product differentiation [21] and are not always able to compete well with the house flora colonizing meat plants, whereby their use often results in losses of desirable sensory characteristics [22]. A culture that performs well in one type of fermented sausage is not necessarily efficient in another type. The use of indigenous strains isolated from spontaneously fermented meats could accentuate artisan-like flavors [21]. Therefore, appropriate cultures have to be selected according to the specific formulation of the batter and technology of fermentation since environmental factors will interact to select a limited number of strains that are competitive enough to dominate the process. The most promising microorganisms for starter cultures are those selected from indigenous microbiota, which are competitive enough to dominate during fermentation, well adapted to the particular product and to the specific production technology, and with high metabolic capacities which can beneficially affect product quality and safety, preserving their typicity [23]. Autochthonous meat starter cultures mainly comprise lactic acid bacteria (LAB) and coagulase-negative cocci (CNC).

### 2.1. Lactic Acid Bacteria

Lactic acid bacteria have a leading role in dry sausage manufacture by acid production and its concomitant pH decrease, affecting both technological properties and microbial stability of the final product. The pH reduction leads to coagulation of fibrillar proteins, which improves the firmness and cohesiveness of final products, enabling slicing [24]; simultaneously, pH drop accelerates the ripening process, which positively affects the moisture of fermented meat products. Along with the production of lactic acid, LAB have a specific enzymatic profile that impacts the taste, aroma, and texture of meat products. They exert lipolytic and proteolytic activity through the action of lipases and proteases which hydrolyze lipids and peptide bonds in proteins, respectively. Lipolysis contributes directly to the typical sensory characteristics of fermented meat sausages while proteolysis enhances their texture and favors the drying process [25]. Some meat LAB have been shown to possess significant nitrate and nitrite reductase activity, even though these activities are more intense in CNC than in LAB [26]. For further reading, Palavecino et al. [27] had extensively reviewed LAB technological properties and safety features.

In keeping with the reclassification of lactobacilli published by Zheng et al. [28], LAB species mainly used as commercial starter cultures are *Latilactobacillus (L.) sakei*, *L. curvatus*, *Lactiplantibacillus (Lc.) plantarum*, *Lc. pentosus*, *Lacticaseibacillus (Lcc.) casei*, *Pediococcus (Pd.) pentosaceus*, and *Pd. acidilactici* [26], while *L. sakei*, *L. curvatus*, and *Lc. plantarum* are the principal species of LAB usually found in spontaneously fermented sausages [29–31]. Members of other LAB genera, such as *Weissella*, *Leuconostoc*, *Lactococcus*, and *Pediococcus* are generally found as minority species [12].

### 2.2. Coagulase-Negative Cocci

The main technological function of CNC is their nitrate reductase activity exerting a definitive effect on typical color development and stabilization of dry sausages. This enzymatic activity relies on the ability of these cocci to reduce nitrate (NO_3_^−^) to nitrite (NO_2_^−^). Red coloration is formed in dry sausages by means of nitrite reduction that leads to nitric oxide (NO), which reacts with myoglobin to form nitrosomyoglobin (MbFeIINO), the compound responsible for the color [32]. Staphylococci can also synthesize nitric oxide from arginine via nitric oxide synthase [33]. Proteolytic and lipolytic activities are among the desirable metabolic characteristics in CNC species [34, 35], releasing several compounds that contribute to the characteristic flavor and texture of fermented meat products, i.e., peptides, amino acids, carbonyls, and volatile substances [34]. Despite being lipolysis mostly performed by endogenous enzymes, CNC lipases can be involved in this process and help to release fatty acids through incomplete *β*-oxidation. Besides, CNC enzymes are able to hydrolyze ester compounds; they also have catalase and superoxide dismutase activity which act as natural antioxidants providing safety [21]. Further technological and safety properties have been extensively described by Palavecino et al. [27].

*Staphylococcus (S.) carnosus* and *S. xylosus* are two CNC species commonly used as commercial starter cultures to assist in color and flavor formation [23]. Many other CNC species prevail in spontaneously fermented sausages, either as dominant: *S. xylosus*, *S. saprophyticus*, and *S. equorum*, or as subdominant: *S. carnosus*, *S. epidermidis*, *S. haemolyticus*, *S. pasteuri*, *S. sciuri*, *S. succinus*, *S. vitulinus*, and *S. warneri*, depending on the type of product [21]. *Kocuria* (*K*.) species are ubiquitous and are highly adapted to the niche of meat fermentation; the species *K. varians* and *K. kristinae* were mainly found in fermented sausage [13].

### 2.3. Yeasts and Molds

The surface of fermented meat sausages is colonized by several yeasts and molds. These microorganisms can play an important role in the quality of the products through the formation of a superficial film which exerts a protective action against both excessive dehydration and the oxidation of the lipid fraction due to oxygen and light [36, 37]. They participate in flavor and aroma development due to the enzymatic activities of their lipases and proteinases [38, 39] and the stabilization of red color of fermented sausages as a result of oxygen depletion [40, 41]. In addition, they may contribute to increased product safety by antagonistic activity against toxigenic molds [42].

Among yeast, there is a clear predominance of the *Debaryomyces* genus on a diverse group comprising *Candida*, *Yarrowia*, *Rhodotorula*, *Pichia*, and *Trichosporon* [43, 44]. *Debaryomyces (D.) hansenii* is the species most frequently and abundantly isolated [36, 39, 45]. The fungal population comprises mainly *Penicillium* and *Aspergillus* genera [46] while other genera such as *Mucor*, *Cladosporium*, *Scopulariopsis*, *Geotrichum*, and *Alternaria* have been found less frequently. *Penicillium* is the most emblematic microbiota of fermented meat sausage surfaces [38], with the prevalence of *Penicillium (P.) nalgiovense*, followed by *P. olsonii*, *P. chrysogenum*, *P. commune*, *P. solitum*, and *P. salamii* [47].

Species such as *D. hansenii*, *P. chrysogenum*, and *P. nalgiovense* are currently used as standard starter cultures. They help to improve and standardize the quality and safety of the final products [43, 46].

## 3. Strain Selection and Design of an Indigenous Starter Culture

Native species that present attractive technological properties can be used to design autochthonous starter cultures that ensure safety and quality of traditional products without altering their typicity. Thus, the introduction of an indigenous starter culture into the production line constitutes an excellent example of the concept “innovation through tradition” (ITT).

The first stage in the designing process of starter culture endogenous to small-scale facilities consists of isolating microorganisms from the niche in which the culture will subsequently be applied. Strains should be selected from the pool of isolates based on technological properties and safety characteristics. Once picked, these isolates should be genetically identified.

General technological properties of interest regarding BAL and CNC were summarized by [48], whereas selection criteria for yeasts and toxicologically safe molds can be found in the literature [43, 46, 49]. Modern approaches for selection of the best strain(s) for autochthonous starter cultures have integrated technical, safety, and health-promoting features [25, 26, 50, 51]. New molecular techniques introduced into the Food Microbiology field complement the studies carried out so far and allow scientists to overcome the limitations of traditional methods [13, 25]. Albeit these advances are helpful for the understanding of meat fermentation, these new technologies are not easily accessible to researchers from emerging countries.

Several authors, around the world, have selected indigenous microbiota to apply as starter cultures in traditional dry sausages; some examples are listed in Table 1. As shown in the table, the species of microorganisms used in different products are the same; however, the variability at strain level is relevant. Therefore, a case-by-case evaluation of a potential bacterial strain to be used as a starter culture must be carried out. Once microorganisms are selected, their compatibility must be checked to determine whether their viability, metabolic activities, and technological features are kept. The desirable scenario is the one where the selected microbes contribute together to the final product without compromising any of their attributes or, even better, when they act additively or synergistically [52, 53]. Furthermore, the selected strains that comprise the designed starter culture should improve the quality and safety whereas keeping or enhancing typical sensory attributes of regional products [20, 21, 50, 54]. Hence, it is advisory to evaluate the sensory profile of the final products, being color, aroma, taste, texture, and overall appearance, the attributes more frequently investigated [20, 53, 55]. Another important aspect to revise is the consistency or robustness of the fermented sausages throughout different production batches, i.e., along a year or a prolonged period, which could guarantee the homogeneity of the product and a good performance of the starter culture [14]. Microbiological and physicochemical characteristics of the products must be monitored throughout the manufacturing process. Regarding the microbiological characteristics, changes in population dynamics and hygienic quality of the products at the end of the ripening stage must be monitored, according to the legislation established in each country or geographical area. Among the physicochemical parameters, pH and *a*_w_ can be evaluated along the process, while salt content, color, texture, fatty acid profile, and volatile compounds can be determined in the final product [14, 20, 22, 53, 56, 57]. Even though these parameters are indeed descriptive, a thorough assessment of the autochthonous starter culture performance can only be achieved when sensory attributes are also examined.

Ferrocino et al. [58] presented a novel work introducing an advance in the understanding of meat fermentation by coupling DNA sequencing metagenomics and metabolomics approaches to describe the microbial function during this process. They proved that a starter culture drastically affected the organoleptic properties of the products by the correlation between the volatilome profile, microbiota, gene content, and consumer acceptability. This evidence highlights the importance of selecting a starter culture to optimize production efficiency and product quality. In addition, metagenomics can be a useful tool when it is integrated to metabolic and sensory analyses giving a better understanding of the functions of starter cultures *in situ* [25]. These authors suggested that it might be probable—in the near future—to know which composition of starter cultures to use in certain technological conditions according to the sensory attributes desired for the product.

An autochthonous starter culture that improves the quality, safety, and homogeneity in traditional sausage manufacture represents a beneficial tool to add value to these products. For fermented foods, microorganisms that are considered autochthonous are often associated with a given area and should be reported as a typical food ingredient. These microorganisms represent a direct link between food and historical and environmental conditions from their original habitat [16]. Narratives about such quality-enhancing functionalities are increasingly being used by starter culture producers, which promote self-styled cultures for “traditional” meat fermentation [59, 60].

## 4. Probiotics

Over the recent decades, the meat industry has focused on enhancing potential health benefits of its products. In this sense, the incorporation of probiotic cultures in traditional meat products could represent an alternative to improve their functional profile. The use of probiotic strains in association with the traditional starter culture has been recognized as a technologically feasible and effective strategy for the development of innovative products [61].

Probiotic cultures are “live microorganisms that, when administered in adequate amounts, confer a health benefit on the host” [62] and comprise both bacteria and yeast. The main genus used as probiotic in meat products is *Lactobacillus*, although other related genera have also been reported, such as *Bifidobacterium*, *Enterococcus*, and *Pediococcus* [63, 64]. Among yeasts, *D. hansenii* has been shown to have probiotic traits, so it could be considered in future innovative developments [65]. The probiotic mechanisms are considered strain-specific and species-specific and some mechanisms might be widespread among commonly studied probiotic genera [66].

Fermented meat products are adequate for the carriage of probiotic bacteria since they do not undergo heat treatment, or else, it is very mild [26, 67]. In addition, it has been postulated that meat matrix protects the survival of probiotic lactobacilli through the gastrointestinal tract [68]. However, the viability of probiotics can be affected by high content of curing salt, low pH, and low water activity of fermented meat products. In this case, technologies such as microencapsulation, which have demonstrated their potential to maintain probiotic viability during processing, storage, and passage through the gastrointestinal tract, can be used [69].

Probiotic cultures in fermented sausages could be autochthonous bacteria with probiotic properties or commercial probiotic strains with documented health-promoting properties. In the first case, the potential probiotic strains can be obtained from fermented sausages by screening microorganisms that possess appropriate physiological requirements and health-promoting properties [70–73]. Even though these potential probiotic strains might be well adapted to the fermentation meat niche, their benefits must be demonstrated in randomized, controlled, or equivalent human trials, either in a heterogeneous or stratified population (based on defined characteristics of host or microbial genomics). Furthermore, dose and genome strain characterization should also be considered [74].

On the other hand, commercial probiotic strains and cultures isolated from human intestinal systems, with documented health-promoting properties, can be used [75]. In this case, the performance of these cultures in the fermentation niche should be evaluated [76]. Regardless of the origin of the probiotic culture, it should not affect the sensory characteristics of the traditional product.

In this sense, the potential use of lactobacillus strains (*Lcc. casei*, *Lcc. paracasei paracasei*, *Lcc. rhamnosus*, and *L. sakei sakei*) isolated from a traditional Italian dry fermented sausage as probiotics was evaluated for Rebucci et al. [77]. Klingberg et al. [78] found that strains *Lc. plantarum* and *Lc. pentosus* originated from fermented meat products were in agreement with the definition of probiotics and their application in the fermented sausages was a success without affecting the flavor of the product. De Pisco and Mauriello [69] reported that some probiotic strains such as *Limosilactobacillus (Lm.) reuteri* ATCC 55730 suffered from the harsh conditions of meat fermented matrix. Rubio et al. [79] found a good performance of the *Lcc. rhamnosus* CTC1679 strain, isolated from the human intestine and with potential probiotic properties, in the manufacture of low-acid fermented sausages “*fuets*.” Subsequent studies showed that *Lcc. rhamnosus* CTC1679 colonized the gastrointestinal tract of healthy volunteers, confirming that it could be delivered as a probiotic in fermented sausages [80]. Ayyash et al. [81] evaluated *Lc. plantarum* KX881772, a new probiotic isolated from camel milk, in semidry fermented camel sausages, finding that this probiotic has promising characteristics for the meat industry.

Regarding microencapsulated probiotic microorganisms, Muthukumarasamy and Holley [82] reported that *Lm. reuteri* ATCC 55730—encapsulated in sodium alginate—was used in fermented meat products. Technological characteristics were not affected, and pH and *a*_w_ values were similar in sausages with encapsulated probiotics and control sausages (free cells), while bacterial viability was also suitable. Sidira et al. [83] evaluated immobilized *Lcc. casei* ATCC 393 on wheat grains in probiotic dry fermented sausages containing reduced or negligible amounts of curing salts. Microbiological and strain-specific multiplex PCR analyses confirmed the levels of *Lcc. casei* ATCC 393 in all samples after 71 days of ripening ranged above the minimum concentration to render a probiotic effect (6 log cfu/g). Ünal Turhan et al. [84] evaluated the production of sucuk with *Lc. plantarum* as starter culture, together with microencapsulated or free cells of *Lcc. rhamnosus* (probiotics strains). Textural, physicochemical, and sensorial properties of sucuk with microencapsulated microorganism were found to be similar to traditional sucuk.

Recently, various studies showed strong evidence that bacterial viability is not an essential requirement to confer health benefits; hence, terms such as paraprobiotics and postbiotics were created to denote health benefits beyond the inherent viability of probiotics. These terms represent new categories of biological response modifier agents [85]. The scientific evidence supports that postbiotics and paraprobiotics possess diverse functional/bioactive properties such as antimicrobial, antioxidant, antihipertensive, and immunomodulatory activities, although mechanisms implicated in most bioactivities of postbiotics and paraprobiotics are not fully understood [66]. Fermented meat products could naturally contain postbiotics and paraprobiotics, but they cannot be controlled, and the amount produced may be insufficient to generate a physiological response *in vivo* [86].

## 5. Antimicrobial and Functional Metabolites

New findings and sophisticated technology applied to the study of microbial metabolites have blurred the line between several definitions. Among the most recent literature, the abovementioned term “postbiotic” is still wide open, mainly when it is referred to as an adjunct substance for food production. According to Moradi et al. [87], any soluble factor (products/metabolic by-products of microbial metabolisms or substances produced by the action of microorganisms on culture/food ingredients) secreted by food-grade microorganisms, or released after cell lysis, during the growth and fermentation in complex microbiological cultures, food or gut, can be considered as such. In the light of this definition, bioactive peptides, exopolysaccharides, bacteriocins, and organic acids could be ascribed to the term postbiotic. These metabolites can exert some benefits to the food or host [88] and could add value to traditional fermented products, applied as an adjunct to living microorganisms—which play a technological role in the manufacture of these products—since postbiotics are more stable and safer for food applications than their producing microorganisms and their viability is not required either during consumption or production [85].

The examples hereafter comprise the most studied bioactive metabolites in fermented meat products. Albeit these metabolites are currently generated *in situ*, this field could be further expanded through the use of cell-free supernatant, from the producing strains, as adjunct agents to indigenous starter cultures.

### 5.1. Antimicrobial Compounds

The main antimicrobial effect responsible for safety of dry sausage is evidently the rate of acidification of the raw meat [89]. Lactic acid bacteria present in the matrix can exert antagonism through competition for nutrients and/or production of several antimicrobial substances such as organic acids (lactic and acetic), carbon dioxide, hydrogen peroxide, diacetyl, ethanol, and bacteriocins. Production of bacteriocins during fermentation of meat plays an important role in enhancing the functional value of meat products, but production of other antimicrobial compounds by specific starter cultures can also be used in fermented sausages.

The role of bacteriocin-producing cultures used as starter cultures—or as adjunct cultures—is twofold: they can contribute to both flavor and food safety, providing fermentation and preservation at the same time. As an illustration, there are commercial cultures such as Bactoferm™ (Chr. Hansen, Denmark), containing pediocin-producing and sakacin-producing strains, used in the manufacture of fermented sausages and dry-cured meat and Holdbac® protective cultures (DuPont Nutrition and Biosciences, USA), containing a mix of bacteriocin-producing strains, used to protect meat, seafood, and dairy products from *Listeria*, yeasts, and molds [90]. Beyond their role as biopreservatives, bacteriocins are gaining credibility as health modulators, due to their ability to regulate the gut microbiota, which is strongly associated with human wellbeing [91].

Several strains of *L. sakei* [70, 92], *L. curvatus* [93–96], *Lc. plantarum* [97–99], and *Pd. acidilactis* [100] isolated from traditional fermented sausages have been reported to produce different bacteriocins, among which curvacins and sakacins are the best known. These bacteriocins are of particular interest due to their high inhibitory activity against *Listeria (Li.) monocytogenes* even though other pathogenic and spoilage microorganisms could also be affected. Extended data regarding the successful use of several bioprotective cultures used in fermented meat products can be found in Aymerich et al. [101] and Oliveira et al. [4].

On the other hand, some authors have reported CNS with antimicrobial activity in preliminary studies, although the bacteriocin production has been mainly described in LAB. In this sense, Lauková et al. [102] found that *S. xylosus* SX S03 1M/1/2 produced a thermostable bacteriocin which could be used as starter culture or meat additive. Sánchez Mainar et al. [103] reported that *S. sciuri* I20-1 exhibited activity against *S. aureus* and *Clostridium (Cl.) botulinum*, owing to the release of its bacteriocin-like substance in a meat model system. The production of bacteriocins by potential CNS starter cultures to fight against *S. aureus* and *Cl. botulinum* in fermented meats could represent a complement to existing antilisterial LAB cultures for the production of safer meat products, in particular when curing agent concentrations are lowered [21].

The use of antagonic microorganisms isolated from traditional meat products may be an important strategy to adequately control toxigenic fungi and protect consumer health from the hazard of exposure to mycotoxins such as ochratoxin A (OTA), aflatoxins (AFs), and cyclopiazonic acid (CPA) [42].

Lactic acid bacteria produce several metabolites able to inhibit fungal growth, which can also participate in mycotoxin degradation and/or removal from contaminated food [104, 105]. Lactobacilli isolated from pork meat and salami showed the ability to inhibit the growth of several molds *in vitro* [106, 107]. Recently, Álvarez et al. [108] reported that *Enterococcus (E.) faecium* SE920, isolated from dry fermented sausages, could be a good candidate to reduce mycotoxin production in dry fermented sausages. In fact, the presence of these strains significantly reduced OTA production of *P. nordicum* in a dry fermented sausage-based medium under conditions simulating sausage ripening.

Antifungal activity was observed in CNS as well. Cebrián et al. [109] reported that meat-borne *S. xylosus* Sx8 exhibited antifungal activity against toxigenic fungi such as *P. nordicum*, *Aspergillus (A.) flavus*, *A. parasiticus*, and *P. griseofulvum*, triggering a significant decrease on mycotoxin accumulation in ham-based medium. However, *S. xylosus* Sx8 antifungal mechanism has not been elucidated yet.

Among yeasts, *D. hansenii* is the most studied species as potential protective culture in fermented sausages because of its predominance in these products [39, 43, 45]. This yeast species has several mechanisms that can tackle fungal growth and mycotoxin production in foods [65] and has been incorporated in the list of qualified presumption of safety (QPS) of the European Food Safety Authority [110]. The use of autochthonous *D. hansenii* strains provided a positive contribution to control the development of spontaneous mold population on fermented Sardinian sausage surface [45] and decrease the growth of ochratoxigenic *P. verrucosum* in dry fermented sausage as a result of the combination of competition for space and nutrients and production of volatile compounds [49]. In addition to the inhibition of the toxigenic mold growth, *D. hansenii* isolated from dry-cured meat has also shown the ability to reduce OTA and AF content in dry fermented sausages against *P. verrucosum* and *A. parasiticus* at transcriptional level [111, 112].

Competition for nutrients and space, together with the production of peptides and proteins with antifungal properties—including a group of small, basic, and cysteine-rich antifungal proteins (AFPs)—is the main mechanism by which some nontoxigenic molds could collaborate in the control of undesirable fungi and improve product safety [113–115]. *Penicillium chrysogenum* isolated from dry-cured meat [116] produces the antifungal protein PgAFP [117] and has shown potential as protective culture by inhibition of *P. griseofulvum* CPA production on dry fermented sausage during ripening [118]. On the other hand, the antifungal protein PgAFP produced *ex situ* was effective in reducing growth of toxigenic *A. flavus* and *P. restrictum* in dry fermented sausages, but its effect was time limited [113]. However, Delgado et al. [119] indicated that the combination of PgAFP and *D. hansenii* could be used as a preventive measure against aflatoxigenic *A. parasiticus* or as a corrective action when this mold has been detected in fermented sausages.

### 5.2. Bioactive Peptides

Bioactive peptides (BP) have been defined as specific protein fragments that have a positive impact on body functions and may influence health. They are encrypted within the sequence of the parent protein and can be released through hydrolysis by proteolytic enzymes. In ripened foods, as dry fermented sausages, hydrolysis can be performed either by endogenous enzymes or by the combined action of endogenous and microbial enzymes [120]. Health benefits of BP are continuously being explored and discovered; hence, there are still controversies regarding their magnitude and significance. Lafarga and Hayes [121] indicated that BP have antimicrobial, antioxidative, antithrombotic, antihypertensive, anticancerogenic, satiety-regulating, and immunomodulatory activities and may affect the cardiovascular, immune, nervous, and digestive systems. Peptides may also be effective in the treatment of mental health diseases, cancer, diabetes, and obesity. Furthermore, many known BP are multifunctional and can present two or more health-promoting activities which may or may not be related [122]. Antioxidant and antihypertensive peptides have been observed in meat products such as Spanish dry-cured ham and dry fermented sausages [123, 124] and Tunisian dry fermented camel sausages [125]. Throughout the manufacturing process, LAB exert an exhaustive proteolysis on the meat matrix. This proteolytic system comprises a cell wall-bound proteinase, peptide transporters, and various intracellular peptidases, including endopeptidases, aminopeptidases, tripeptidases, and dipeptidases [126], which contribute to the generation of small peptides and free amino acids [127]. Although it is known that the degradation of main myofibrillar and sarcoplasmic proteins took place in meat products [128, 129], peptides generated in dry fermented meat products have been less studied. Recently, a study from Mora et al. [124] reported that intense proteolysis occurred during processing due to the action of peptidases from muscle and LAB using a peptidomic approach.

Regarding what concerns this review, the use of autochthonous strains as potential bioactive peptide releasers has been assessed by few authors. Among three strains isolated from a camel meat sausage obtained by fermentation with endogenous microflora, Mejri et al. [125] found that the batch inoculated with *S. xylosus* and *Lc. plantarum* (10^7^ cfu/g) had higher antioxidant and antihypertensive capacities than the noninoculated sausages. Fractions with molecular weights below 3 kDa showed the highest antioxidant and antihypertensive capabilities in comparison with fractions above 3 kDa, which were observed at the end of ripening. Analysis of these fractions by RP-HPLC-ESI-Q-TOF-MS/MS allowed the identification of 13–22 peptides with a number of amino acids that ranged from 4 to 23. These peptides shared many common features with other antioxidant and antihypertensive peptides. Escudero et al. [123] and Mora et al. [124] stated that low molecular weight peptides (<3 kDa), contributing to the development of flavor in dry fermented meat products, could also exert antihypertensive activity.

A study focused on health-promoting benefits of fermented camel sausages conducted by Ayyash et al. [81] showed that the probiotic bacteria *Lc. plantarum* KX881772—isolated from camel milk—played a promising role at imparting functional characteristics to these sausages. The release of bioactive peptides with greater antioxidant capacities, ACE inhibition, and cytotoxicity (antitumoral) activity than the control was due to a high degree of hydrolysis achieved with the addition of the probiotic strain to the camel sausage.

Fernández et al. [130] presented the joint use of an autochthonous starter culture composed by the strains *Pd. acidilactici* MS200 and *S. vitulus* RS34, together with the protease EPg222 (purified from *P. chrysogenum* Pg222, which was isolated from dry-cured meat products). Their study showed that angiotensin-I-converting enzyme (ACE) inhibitory and antioxidant compounds were released during the ripening process of dry fermented sausages. Even though the main proteolytic effect had been attributed to the enzyme, they suggested the combined use of the autochthonous starter and the enzyme since both had proved to give the best organoleptic and hygienic profiles in previous studies [56].

## 6. Reduction of Salt and Fat

Sodium chloride is an important ingredient in processed meat due to its preservative properties, its capacity to affect taste and improve product flavor, and its functional capacity to solubilize myofibrillar proteins, which is necessary in order to enhance adhesion and cohesiveness in processed meat products [131]. Granulated fat contributes to the continuous release of moisture—a process necessary for the fermentation and flavor development [132]. Fat contributes to the texture, mouthfeel, juiciness, and lubricity and constitutes a source of essential fatty acids and fat soluble vitamins [133].

In recent years, increased concerns about the potential health risks associated with the consumption of food with high content of sodium and fat have led the meat industry to develop new formulations or modify traditional food products to contain less sodium and fat [131, 133–135]. These new formulations confer functionality to fermented meat products that could be boosted with the use of indigenous starter cultures.

Several strategies to reduce salt content could be applied to small manufacturing industries; the simplest is the direct reduction of salt content, which is only possible in small proportions due to technological contributions of NaCl. Another strategy consists of the partial substitution of NaCl for salts such as KCl, CaCl_2_, or MgCl_2_. In this sense, KCl is the most used substitute in fermented meat products and exerts an inhibitory effect on muscle proteases similar to NaCl; but their main limitation is the metallic flavor [136]. Another alternative is to modify the salt size; however, the salt size manipulation in meat products has not been shown yet [131].

Laranjo et al. [135] analysed the effect of salt reduction on Portuguese traditional dry-cured sausages. They found that a reduction of 50% of salt did not affect the safety of the product but the flavor and the texture. Later, they evaluated the same salt reduction in traditional blood dry-cured sausages, which in this case had a positive effect on product acceptance by the panelists without compromising the microbiological stability or fatty acid profile of dry-cured sausages, but biogenic amine levels increased [137]. Therefore, they propose to complement the reduction of salt with the use of starter cultures to minimize the levels of biogenic amines and promote the development of flavor.

De Almeida et al. [138] evaluated the performance of autochthonous starter cultures in a low-sodium fermented sausage model, containing 1% NaCl, 0.25% KCl, and 0.25% CaCl_2_. They found a combination of strains with peptidogenic potential (*E. mundtii* CRL35+*S. vitulinus* GV318) and another combination with influence on the production of amino acids (*L. sakei* CRL1862+*S. vitulinus* GV318). This release of amino acids and small peptides is frequently related to the taste of processed meat.

Dry fermented sausages are one of the most difficult meat products as far as fat reduction is concerned since excessive fat reduction leads to unacceptable appearance and undesirable changes in texture [133]. While removing back fat from the batter is a simple way to perform this reformulation, limitations are given by the reduction of sensory and technological quality of product [76]. The replacement of animal fats with plant oil sources in processed meat products affects the drying process and presents problems such as inadequate drying, binding, and retention of liquid oils [139, 140].

Olivares et al. [141] reported that with a reduction of fat in the raw material of around 15%, using controlled ripening conditions and slow fermentation process, they obtained fermented sausages with high consumer acceptability. Then, they studied the effect of fat content (10, 20, and 30%) and ripening time and found that fat reduction decreased lipolysis, lipid oxidation, and lipid-derived volatile compounds during processing while the volatile compounds generated from bacterial metabolism increased, although only in the first stages of processing. The consumer preference in aroma and overall quality of high (30%) and medium (20%) fat sausages was related to the aroma compounds, and these contents of fat were not differentiated by consumers [140].

In terms of salt and fat reductions together, Rubio et al. [142] evaluated potential starter lactobacilli strains in low-acid fermented sausages (*fuets*), with reduced salt and fat content. In this study, 25% of NaCl was substituted with KCl; and the fat pork was reduced a lower proportion than usual. They achieved a reduction in salt and fat (25% and 52%, respectively) without detrimental effects on the sensory quality of the fuets. Mora-Gallego et al. [143] have shown that even a reduction of 70% of fat in nonacid fermented sausages resulted in satisfactory overall sensory quality of fuet-type sausages. Mora-Gallego et al. [133] found that the reduction of fat proportion (from 20% to 10%) and salt (from 2.5% to 1.5%) had good consumer acceptability in dry fermented sausage type *fuet*, but these simultaneous reductions led to an increase in *a*_w_. The increase of *a*_w_ was compensated by an addition of 0.64% KCl, which does not negatively affect the consumer acceptability. *D. hansenii* P2—isolated from naturally fermented sausages—was used as starter in the production of dry fermented sausages manufactured varying salt and/or pork back fat content and sensory analysis showed that yeast inoculation improved the aroma and taste quality when fat or salt reductions were done [144]. The contribution of inoculation to the reformulated dry sausages was attributed to the increase in aroma compounds derived from amino acid degradation and ester activities which increased the perception of fruity and cured aroma [145]. Nevertheless, yeast inoculation did not show a clear effect when salt and fat reduction was carried out together.

## 7. Additional Support Strategies

### 7.1. Quality Product Registers

In many economic regions, there exist quality policies aimed at protecting the names of specific products to promote their unique characteristics, linked to their geographical origin as well as traditional know-how [146]. Fermented meat products, namely, dry sausages, fall into this set of products. The geographical indication (GI) is a sign used on products that have a specific geographical origin and possess qualities or a reputation that are due to that origin [147]. In the case of foodstuffs and wine, GI comprised Protected Designation of Origin (PDO) and Protected Geographical Indication (PGI). As stated by the WIPO: “In order to function as a GI, a sign must identify a product as originating in a given place. In addition, the qualities, characteristics, or reputation of the product should be essentially due to the place of origin. Since the qualities depend on the geographical place of production, there is a clear link between the product and its original place of production.” From a wider perspective, these quality schemes can play a special role in promoting sustainable rural development, improving farm income and opening new export potential.

Geographical indications have to be requested to the corresponding national or international authorities; this bureaucratic paperwork could be time-consuming; there are legal aspects to be fulfilled; and some extra taxes might be applied on the products depending on the legislation. However, the benefits associated with these concepts justify these actions. Having a GI could broaden the marketing boundaries of artisanal dry sausages. Fermented meat products can be added as local craft elements on touristic routes, embracing the current tendency of synergy between tourism, culture, and gastronomy. Furthermore, GIs comprise knowledge and skills passed on from generation to generation, helping to protect local heritage.

### 7.2. Innovation through Tradition

De Massis et al. [148] conceptualize a new strategic approach, called “innovation through tradition,” which identifies the elements that enterprises possess to profit from leveraging the past, thus combining tradition and innovation into new products. Within the food sector, consumers' needs tend to be satisfied by offering products being able to balance tradition and innovation [149]. The need for innovation in dry sausages, mainly in traditional small-scale manufacture, appears as a big challenge.

Small- and medium-sized enterprises (SMEs) need to find ways to maintain and increase their niche markets under the pressure of rapid technological changes while the production rationale remains as an art. On the one hand, there is a significant development of codified knowledge and on the other traditional manufacturing practices and skilled sausage makers with a colorful history. Codified knowledge is explicit and consists of facts, theories, and principles that are codified in research journals, taught in universities and recorded in industries [150]. Tacit knowledge is widely held by individuals and not able to be readily expressed as it is skill, know-how, and expertise [151]. Traditional recipes which connected early European immigrants with their homeland are very common in this target segment, mainly in those countries known as the “new world” (e.g., Canada, Australia, USA, and Latin American communities). Particular raw ingredient compositions are processed according to these recipes that have been passed down from one generation to another. Although certain problems of product quality variations are recorded, unique flavors from small artisanal firms producing dry sausages are very popular among local consumers.

At this point, one of the critical issues in the heart of leveraging traditional sausage manufacturing with scientific developments consists of finding the ways for doing so successfully. Building on this reasoning, merging tacit and codified knowledge seems to be an imperative for scholars if they aim to leverage the potential of small artisanal dry sausage local firms. Science, technology, economy, culture, ecosystem, and interorganizational synergies are, at least, main aspects for strategies related to territorial networks, production, and innovation. Promoting multilevel connection among firms, university, and government appears as a master hint for leveraging sausage manufacturing clusters which are not only sectoral but also geographical. In this sense, there is a need for a change in focus to multidimensional and specific-context knowledge based on firm-by-firm analysis. Moreover, this new innovation perspective needs a broader picture than a microeconomic level; only a micro-meso-macro approach may lead to local development as a collective and cooperation-based learning process.

## 8. Conclusions

Microbial cultures specifically chosen for any meat fermented product should help to control undesirable native flora and give a uniform quality to food in every batch. Technological support to incorporate these cultures into the production process might become an indispensable part of innovation and creativeness for small-scale facilities. A great combination of selected microbial cultures could be designed to offer improved and/or preserved sensorial attributes among which flavor, overall appearance, texture, and stability of the final product are of paramount importance. Even though the introduction of biotechnology could represent a competitive advantage for small-scale facilities, typical sensory properties need to be preserved, particularly when the product is perceived as rooted in a cultural past that has been dominated by craftsmanship and it is valued as such.

Besides safety and health, the meat industry aims at innovation by generating a superior perceived quality while focusing on traditional flavors. Consumers' acceptability is the master key for successful product innovation. Herein, several technological processes associated to indigenous microbial cultures aimed at improving the quality of artisanal fermented meat products were presented as potential strategies for small-scale facilities. The authors are aware of the challenge that these implementations would entail considering that microbial strains should be isolated from each facility. Nonetheless, it might be a reasonable enterprise in the light of the benefits that may be gained from these “microscopic factories.” Facing the next decade and beyond implies moving forward to a more healthy, sustainable, and fair food system. Within this context, traditional foods should be used to valorize niche productions, uphold small-scale producers, and deepen the ties between food and places, thereby safeguarding the territories and the biodiversity of autochthonous cultures and raw matter.

## Figures and Tables

**Table 1:** Indigenous starter cultures evaluated in the production of dry fermented sausages.

Product	Strains or combinations of strains	Inoculum (cfu/g)	Processing place	References
Traditional dry fermented sausage	*L. sakei* F08F202+*S. equorum* F08bF15+*S. succinus* F08bF19	1 × 10^6^	French traditional processing unit	[152]
Greek fermented sausage	*L. sakei* 8416 *L. sakei* 4413 *L. sakei* 8426 *Lc. plantarum* 7423 *L. curvatus* 8427	2–5 × 10^7^	N.E.	[20]
Basilicata fermented sausage	*L. sakei* DBPZ0062+*S. equorum* DBPZ0241	1 × 10^6^	Artisanal industry	[22]
Iberian dry fermented sausage	*Pd. acidilactici* MC184+*S. vitulinus* RS34 *Pd. acidilactici* MS198+*S. vitulinus* RS34 *Pd. acidilactici* MS200+*S. vitulinus* RS34	5 × 10^7^	Two different industries	[153]
Galician chorizo	*L. sakei* LS131+*S. equorum* SA25 *L. sakei* LS131+*S. epidermidis* SA49 *L. sakei* LS131+*S. saprophyticus* SB12	1 × 10^6^–10^7^	N.E.	[154]
Slow dry-cured fermented sausages	*D. hansenii* M4+C-P-77S bactoferm^1^ *D. hansenii* P2+C-P-77S bactoferm	1 × 10^6^	N.E.	[155]
Traditional dry sausage from Chaco	*L. sakei* 487+*S. vitulinus* C2 *L. sakei* 442+*S. xylosus* C8	1 × 10^6^	Small-scale facility	[14, 53]
Chinese fermented dry sausage	*Lc. plantarum* CMRC6 *L. sakei CMRC15*	1 × 10^7^	Food pilot plant	[128]
Salami	*P. nalgiovense* ITEM 15292*+*Startec TCSD1^2^ *P. salamii* ITEM 15302+Startec TCSD1 (surface inoculation for fungi)	1.48 log cfu/cm^2^ 1.93 log cfu/cm^2^	Two different industries	[156]
Fermented camel sausage	*Lc. plantarum+S. xylosus* *Lc. pentosus+S. xylosus*	1 × 10^7^	N.E.	[125]
Horse meat sausage	*L. sakei* 121	1.5 × 10^6^	Small-scale facility	[8]
Sardinian fermented sausages	(*Lc. plantarum* PC23*+S. xylosus* SA23)*+D. hansenii* Ca3 *D. hansenii* Ca3 (surface inoculation for yeast)	1 × 10^6^ 5.9-8.3 log cfu/g	Farm scale	[45]
Chinese dry fermented sausage	*Lc. plantarum* R2 *Lc. plantarum* R2*+S. xylosus* A2	1 × 10^7^	N.E.	[157]

^1^C-P-77S bactoferm: starter culture containing *Lc. pentosus* and *S. carnosus* (Chr. Inc., Hansen, Denmark). ^2^Startec TCSD1: commercial starter culture, Tec-Al S.R.L. N.E.: not specified.
